# Tricuspid repair: short and long-term results of suture annuloplasty techniques and rigid and flexible ring annuloplasty techniques

**DOI:** 10.1186/s13019-024-02640-y

**Published:** 2024-03-27

**Authors:** Ufuk Türkmen, Tezcan Bozkurt, Sertan Özyalçın, Ilknur Günaydın, Sadi Kaplan

**Affiliations:** 1 Department of Cardiovascular Surgery, Hitit University Faculty of Medicine, Corum, Turkey; 2 Department of Cardiovascular Surgery, Manisa City Hospital, Manisa, Turkey; 3 Department of Cardiovascular Surgery, Ankara Etlik City Hospital, Ankara, Turkey; 4 Department of Cardiovascular Surgery, Ankara Bilkent City Hospital, Ankara, Turkey; 5 Department of Cardiovascular Surgery, Hacettepe University Faculty of Medicine, Ankara, Turkey

**Keywords:** Tricuspid valve repair, Annuloplasty Techniques, Functional tricuspid regurgitation, Systolic pulmonary artery pressure, NYHA Functional Capacity

## Abstract

**Background:**

Functional tricuspid regurgitation may arise from left heart valve diseases or other factors. If not addressed concurrently with primary surgical intervention, it may contribute to increased morbidity and mortality rates during the postoperative period. This study investigates the impact of various repair techniques on crucial factors such as systolic pulmonary artery pressure (SPAP), tricuspid valve regurgitation, and New York Heart Association (NYHA) functional capacity class in the postoperative period.

**Materials and methods:**

From April 2007 to June 2013, 379 adults underwent open-heart surgery for functional tricuspid regurgitation. Patients were categorized into four groups: Group 1 (156) with De Vega suture annuloplasty, Group 2 (60) with Kay suture annuloplasty, Group 3 (122) with Flexible Duran ring annuloplasty, and Group 4 (41) with Semi-Rigid Carpentier-Edwards ring annuloplasty. Demographic, clinical, operative, and postoperative data were recorded over a mean follow-up of 35.6 ± 19.1 months. Postoperative SPAP values, tricuspid regurgitation grades, and NYHA functional capacity classes were compared among the groups.

**Results:**

No statistically significant differences were observed among the groups regarding age, gender, preoperative disease diagnoses, history of previous cardiac operations, or echocardiographic characteristics such as preoperative ejection fraction, SPAP, and tricuspid regurgitation. Hospital and intensive care unit length of stay and postoperative complications also showed no significant differences. However, patients in Group 3 exhibited longer Cardio-Pulmonary Bypass duration, cross-clamp duration, and higher positive inotrope requirements. While the mortality rate within the first 30 days was higher in Group 1 compared to the other groups (*p*: 0.011), overall mortality rates did not significantly differ among the groups. Significant regression in functional tricuspid regurgitation and a notable decrease in SPAP values were observed in patients from Group 3 and Group 4 (*p*: 0.001). Additionally, patients in Group 3 and Group 4 showed a more significant reduction in NYHA functional capacity classification during the postoperative period (*p*: 0.001).

**Conclusion:**

Among the repair techniques, ring annuloplasty demonstrated superiority in reducing SPAP, regressing tricuspid regurgitation, and improving NYHA functional capacity in functional tricuspid regurgitation repairs.

## Introduction

There exists a general consensus regarding the surgical management of functional tricuspid valve regurgitation (FTR) [1, 2]. Surgical approaches to FTR encompass a range of methods, including semicircular suture annuloplasty (De Vega), annular plication (Kay method), annular ring annuloplasty techniques, and valve replacement. Annuloplasty rings, prostheses affixed to the natural valve annulus, play a crucial role in correcting annulus dilation, reshaping it, facilitating complete leaflet closure during systole, and complementing valve repair [3]. The evolution of annuloplasty rings initiated with Carpentier’s groundbreaking application of the first rigid ring procedure in humans, followed by the introduction of flexible ring materials by Duran and Cosgrove. Notably, flexible rings offer a significant advantage over rigid ones by allowing physiological movement of the tricuspid annulus [4, 5]. In light of the absence of a definitive ideal repair method, the anticipation is for new approaches to emerge, contributing to the diversification of future FTR operations.

This study aims to identify the most suitable method by analyzing the clinical and echocardiographic outcomes associated with various annuloplasty techniques applied for FTR, both pre and post-surgery.

## Material and method

### Study design and patient population

#### Methods

Retrospectively, we analyzed the data of 735 patients who underwent open-heart surgery at the Cardiovascular Surgery Clinic of Türkiye Yüksek İhtisas Training and Research Hospital between April 2007 and June 2013. These patients received tricuspid valve intervention for FTR. Exclusion criteria include patients under 18 years of age, those who underwent surgery due to dilated cardiomyopathy, endocarditis, rheumatic or organic tricuspid valve disease, tricuspid stenosis, those who underwent emergency heart surgery, and those who developed renal failure in the postoperative period. Patients with a transvalvular permanent pacemaker during valve repair and those requiring a transvalvular permanent pacemaker in the postoperative period were also excluded. The final study cohort comprised 379 patients after excluding those with incomplete data. The patients were non-randomly categorized into four groups based on the specific annuloplasty method applied to the tricuspid valve.

#### Groups

Group 1 (De Vega annuloplasty, *n* = 156).

Group 2 (Kay annuloplasty, *n* = 60).

Group 3 (Flexible Duran ring annuloplasty, Medtronic, Minneapolis, Minnesota, *n* = 122).

Group 4 (Semi-Rigid Carpentier-Edwards ring annuloplasty, Edwards Lifesciences, Irvine, Calif, *n* = 41).

#### Data collection

Demographic, clinical, and operative data, along with postoperative follow-up results, were sourced from patient files, hospital archives, and clinical computer records. The New York Heart Association (NYHA) classification determined the preoperative and postoperative functional capacities of all patients. Transthoracic echocardiography findings were compared among the four groups for preoperative, early postoperative, and long-term assessments. Analysis parameters included the postoperative reduction in systolic pulmonary artery pressure (SPAP), regression in FTR grade, regression in NYHA functional capacity, and other clinical conditions.

#### Postoperative data

The early postoperative data collected within the first month after surgery includes 362 patients, excluding 17 patients who succumbed within the initial 30 days. In order to determine the postoperative 5-year survival, inquiries were conducted through the patient registration system. The long-term postoperative data of 351 patients are based on the most recent examinations conducted in our clinic.

### Surgical procedure

Following the induction of general anesthesia, routine intraoperative assessments were conducted on patients. A 7 F catheter was placed through the right internal jugular vein post-intubation. Electrocardiographic data obtained via five electrodes on the back were continuously monitored. Standard median sternotomy was performed on all patients, utilizing an air-powered saw for those with a history of prior open-heart surgery. Access to the heart was achieved through a combination of blunt and sharp dissection. Anticoagulation was attained with heparin at a dose of 300 IU/kg, and its effects were monitored using activated clotting time (ACT), with additional heparin administered as necessary to maintain an ACT above 400 s.

Aortic cannulation was uniformly performed using a 24 F (curved tip) cannula. Bicaval venous cannulation (32 F from the right atrial appendage to the superior vena cava and a 36 F cannula from the right atrial wall to the inferior vena cava) was implemented to initiate cardiopulmonary bypass. Rectal temperature was monitored, and upon initiating bypass, the patient’s temperature was reduced to 30–32 °C. Continuous perfusion with non-pulsatile flow commenced following aortic cross-clamping. Cold cardioplegia, administered via both antegrade and retrograde routes, induced diastolic arrest. Myocardial protection was further ensured through topical hypothermia using a 0.9% NaCl solution at + 4 °C. A membrane oxygenator was employed in all cases, maintaining hematocrit between 20% and 25% during cardiopulmonary bypass (CPB). Pump flow was maintained between 2 and 2.2 L/min/m² with non-pulsatile flow, and average arterial pressure was sustained at 50–60 mm/Hg throughout the cross-clamp period. Valve repair techniques were chosen based on the surgeon’s discretion.

For De Vega annuloplasty, the “annulus constriction” method involved passing both ends of a 2/0 polypropylene pleated suture through the anteroseptal commissure, proceeding from the annulus. The suture traversed the annulus intermittently, reaching the posteroseptal commissure. Both ends were then passed through a new pledget and tightened to a specific extent, adjusted using a 50 ml syringe with a ring scale or manually to match the width of two fingers. Kay annuloplasty utilized two concentric 2/0 Ethibond sutures, effectively obliterating the posterior leaflet and creating a bicuspid AV valve. Manual adjustment to a 50 ml syringe or two-finger width assessed the adequacy of the repair. For ring annuloplasty, the ring size was determined through measurement, attached to the annulus with 2/0 Ethibond sutures passed in a U-shape parallel to the annulus. Sutures avoided the annulus of the septal leaflet to prevent damage to the AV node and conduction system, ensuring correct positioning within the tricuspid ring. Right ventricular filling with a syringe concluded the procedure, and valve function was assessed.

Closure of the right atriotomy utilized a 5/0 polypropylene suture. Cardio-pulmonary bypass (CPB) was terminated upon removal of the cross-clamp on the ascending aorta, following adequate heart warming and contraction strength.

### Echocardiographic monitoring

In the patient follow-up, transthoracic echocardiography (Vivid 7 Dimension, GE Medical Systems, Horten, Norway) was conducted utilizing a 2.5-3.5 MHz transducer. Standard M-mode measurements were performed in accordance with the guidelines of the American Society of Echocardiography. Ejection fraction (EF) calculations employed the modified Simpson method based on two-plan apical (2 and 4-cavity) images [6].

Evaluation of FTR utilized color Doppler on the apical four-cavity image. Continuous wave Doppler was employed to obtain the peak tricuspid regurgitant flow velocity, and the right ventricle-right atrium peak pressure gradient was calculated using the modified Bernoulli equation. The systolic pulmonary artery pressure (SPAP) value was determined by adding the right atrial pressure to this measurement [7].

The grading of FTR was categorized into four classes based on the distance of the regurgitation jet flow from the cardiac apex in a four-cavity view: 1° TI for less than 15 mm, 2° TI for 15–30 mm, 3° TI for 30–45 mm, and 4° TI for more than 45 mm [8].

### Statistical analysis

The data acquired in this study underwent analysis using the IBM Statistical Package for the Social Sciences (SPSS for Windows, Armonk, NY, Version 22.0.0; IBM, 2013, IL). Continuous variables were expressed as mean ± standard deviation, while categorical variables were presented as numbers and percentages. Cross-tabulations were performed using Chi-square and Fisher’s exact chi-square tests. For parametric data, One-way Analysis of Variance (ANOVA) was employed, while non-parametric data were assessed using the Kruskal-Wallis test.

To evaluate preoperative, early, and long-term results within each group, Repeated Measures ANOVA was utilized for parametric data, and the Friedman test was applied for non-parametric data.

In instances where differences were observed following variance analyses, post hoc assessments were conducted. Specifically, Kruskal-Wallis and Friedman tests, along with post hoc multiple comparison tests such as Bonferroni, were employed to identify specific pairs exhibiting significant differences.

A *p*-value of less than 0.05 was deemed statistically significant in all analyses.

## Results

### Demographic characteristics and comorbidities

Table 1 presents the demographic characteristics and comorbid diseases of the patients. The mean age of the patients was 51.6 ± 13.3 years, with no statistically significant difference in mean ages observed among the groups (*p*: 0.981). Gender distribution similarly did not show a statistically significant difference between the groups (*p*: 0.099). The prevalence of hypertension (44.6%), diabetes (27.7%), chronic obstructive pulmonary disease (10.3%), and haemodialysis-dependent renal failure (1.6%) did not significantly differ among the groups (*p* > 0.05). However, there was a significantly higher incidence of a history of atrial fibrillation (AF) in Group-1 and Group-2, and a significantly higher incidence of previous cerebrovascular events (CVE) in Group-2 and Group-4 compared to the other groups (*p*: 0.005).

**Table 1:** Preoperative comparison of patients regarding demographics, comorbidities, previous operations, echocardiographic findings, NYHA.

Parameters		Group-1 (*n* = 156)	Group-2 (*n* = 60)	Group-3 (*n* = 122)	Group-4 (*n* = 41)	Total (*n* = 379)	Test Statistics
**Demographic data and comorbidities**	**Age (years)**	52.2 ± 12.6	51.9 ± 13.3	50.9 ± 14.5	50.8 ± 12.8	51.6 ± 13.3	p:0.981 *
	**Female**	117 (75.0%)	44 (73.3%)	79 (64.8%)	24 (58.5%)	264 (69.7%)	*p*:0.099**
	**HT**	63 (40.4%)	26 (43.3%)	58 (47.5%)	22 (53.7%)	169 (44.6%)	*p*:0.399**
	**DM**	48 (30.8%)	18 (30.0%)	26 (21.3%)	13 (31.7%)	105 (27.7%)	*p*:0.295**
	**COPD**	15 (9.6%)	8 (13.3%)	12 (9.8%)	4 (9.8%)	39 (10.3%)	*p*:0.869**
	**CRF**	3 (1.9%)	1 (1.7%)	2 (1.6%)	0 (0.0%)	6 (1.6%)	*p*:0.854**
	**AF**	102 (65.4%)	48 (80.0%)	55 (45.1%)	18 (43.9%)	223 (58.8%)	p:0.001**
	**CVE**	3 (1.9%)	6 (10.0%)	2 (1.6%)	4 (9.8%)	15 (4.0%)	*p*:0.005**
**Previous operations**	**CMC**	6 (3.8%)	3 (5.0%)	3 (2.5%)	0 (0.0%)	12 (3.2%)	*p*:0.381 **
	**MVR**	4 (2.6%)	2 (3.3%)	3 (2.5%)	0 (0.0%)	9 (2.4%)	
	**AVR**	2 (1.3%)	0 (0.0%)	0 (0.0%)	1 (2.4%)	3 (0.8%)	
	**ASD**	0 (0.0%)	1 (1.7%)	0 (0.0%)	1 (2.4%)	2 (0.5%)	
**ECHO measurements**	**EF %**	52.8 ± 8.7	51.6 ± 9.8	51.6 ± 9.5	52.3 ± 7.5	52.2 ± 9.0	*p*:0.803 *
	**SPAP**	50.8 ± 11.5	52.0 ± 12.7	49.5 ± 7.8	54.7 ± 12.4	51.0 ± 10.9	*p*:0.148 *
**FTR** **Degree**	**2**	49 (31.4%)	19 (15.6%)	45 (36.9%)	9 (7.4%)	122 (32.2%)	*p*:0.508**
	**3**	100 (42.2%)	39 (16.5%)	70 (29.5%)	28 (11.8%)	237 (62.5%)	
	**4**	7 (4.5%)	2 (3.3%)	7 (5.7%)	4 (9.7%)	20 (5.3%)	
	**Mean ± Std. D.**	2.7 ± 0.5	2.7 ± 0.5	2.7 ± 0.6	2.9 ± 0.5	2.7 ± 0.5	*p*:0.303 *
**Mitral Valve Structure**	**NON-Mitral Valve**	0 (0.0%)	0 (0.0%)	14 (11.5%)	12 (29.3%)	26 (6.9%)	p:0.001 **
	**MVI**	32 (20.5%)	11 (11.6%)	37 (30.3%)	15 (15.8%)	95 (25.1%)	
	**MVS**	124 (48.1%)	49 (19.0%)	71 (27.5%)	14 (5.4%)	258 (68.1%)	
**Functional NHYA** **Class**	**2**	23 (14.7%)	12 (16.9%)	31 (25.4%)	5 (7.0%)	71 (18.7%)	p:0.001 **
	**3**	112 (43.1%)	42 (16.2%)	84 (32.3%)	22 (8.5%)	260 (68.6%)	
	**4**	21 (13.5%)	6 (10.0%)	7 (5.7%)	14 (29.2%)	48 (12.7%)	
	**Mean ± Std. D.**	3.0 ± 0.5	2.9 ± 0.5	2.8 ± 0.5	3.2 ± 0.6	2.9 ± 0.5	p:0.001 *

HT: Hypertension; DM: Diabetes mellitus; COPD: Chronic obstructive pulmonary disease; CRF: Chronic renal failure; AF: Atrial fibrillation, CVE: Cerebrovascular event, CMC: Closed mitral commissurotomy, MVR: Mitral valve replacement, AVR: Aortic valve replacement, ASD: Atrial septal defect, ECHO: Echocardiography, EF: Ejection Fraction; SPAP: Systolic Pulmonary Artery Pressure, MVS: Mitral valve stenosis, MVI: Mitral valve insufficiency, FTR: functional tricuspid valve regurgitation, NYHA: New York Heart Association, Mean: Mean, Std. S.: Standard deviation,

*Kruskal-Wallis test **Chi-Square test

*Kruskal-Wallis test **Ki-Kare testi

### Preoperative evaluations

The comparison of preoperative EF percentage and SPAP values among the different operation groups showed no significant differences (*p* > 0.05). When evaluating the degree of FTR detected by echocardiography in the preoperative period as a categorical variable and its distribution among the groups, no significant difference was found (*p*: 0.508) (Table 1). However, a statistically significant difference was observed in the preoperative New York Heart Association (NYHA) functional capacity classification among the operation groups (*p*: 0.001). Group 4 had a higher proportion of NYHA classes 3 and 4, while the other groups had higher proportions of NYHA classes 2 and 3 (Table 1).

### Mitral valve disease and surgical history

A statistically significant difference was found in the presence or absence of mitral valve disease detected by echocardiography in the preoperative period as a categorical variable and its distribution among the four different operation groups (*p*: 0.001) (Table 1). Among all groups, 258 (68.1%) patients were diagnosed with mitral stenosis preoperatively. Although 26 (6.9%) of the 379 patients included in the study had previously undergone open-heart surgery for closed mitral valvotomy, mitral valve replacement (MVR), aortic valve replacement (AVR), and atrial septal defect (ASD) repair, there was no statistically significant difference between the groups (*p*: 0.381) (Table 1).

### Accompanying procedures in tricuspid valve groups

Table 2 outlines the additional operations performed alongside FTR operations in the four tricuspid valve groups. The most common accompanying procedure was mitral valve operation (93.1%). Other procedures included aortic valve operation in 74 cases (19.5%), ASD repair in 51 cases (13.5%), coronary artery bypass grafting (CABG) in 39 cases (10.3%), ascending aorta surgery in 13 cases (3.4%), and ventricular septal defect (VSD) repair in 3 cases (0.8%).

### Frequency of additional procedures

Analyzing the frequency of these procedures performed alongside FTR surgery in the four groups revealed significant differences in mitral and aortic valve operations, ASD repair, and CABG operations (Table 2). Mitral valve operations were predominantly performed in Group 1 and Group 2, while aortic valve operations were more common in Group 3 (31.1%). CABG and ASD repair were more frequently performed in Group 4 (22.0% and 29.3%). There were no statistically significant differences in the distribution of patients who underwent ascending aortic surgery and VSD repair across the four groups (*p* > 0.05).

### Operative duration and positive inotropes

The duration of cardiopulmonary bypass (CPB) and cross-clamping did not significantly differ among the four operation groups (*p*: 0.001). However, the CPB and cross-clamping time in Group 3 were significantly longer than in Groups 1 and 2, with no significant differences observed in Group 4 (Table 2). Evaluation of the need for positive inotropes after cross-clamping revealed a significant difference among the four groups (*p*: 0.012), with Group 3 demonstrating a higher positive inotropes requirement than the other groups.

### Follow-up and mortality

The average follow-up period in the study, employing four different tricuspid valve annuloplasty techniques, was 35.6 ± 19.1 months (Table 2). The mortality rate within the first 30 days was higher in Group-1 patients compared to the other groups (*p*: 0.011). However, there were no differences in overall mortality rates among the groups (*p*: 0.453). Additionally, there were no significant differences in 5-year survival rates, with an overall 5-year survival rate of 87.9% (%*p*: 0.400) (Table 2).

**Table 2:** Comparison of the groups regarding simultaneous surgeries, CPB time, cross-clamp time and mortality data

Parameters		Group-1 (*n* = 156)	Group-2 (*n* = 60)	Group-3 (*n* = 122)	Group-4 (*n* = 41)	Total (*n* = 379)	Test Statistics
**Simultaneous Surgeries**	**Mitral valve surgery**	156 (100.0%)	60 (100.0%)	108 (88.5%)	29 (70.7%)	353 (93.1%)	*p*:0.001*
	**Aortic valve surgery**	23 (14.7%)	7 (11.7%)	38 (31.1%)	6 (14.6%)	74 (19.5%)	*p*:0.013 *
	**ASD repair**	12 (7.7%)	6 (10.0%)	21 (17.2%)	12 (29.3%)	51 (13.5%)	*p*:0.001 *
	**Coronary artery bypass**	11 (7.1%)	4 (6.7%)	15 (12.3%)	9 (22.0%)	39 (10.3%)	*p*:0.027 *
	**Ascending aortic surgery**	4 (2.6%)	4 (6.7%)	3 (2.5%)	2 (4.9%)	13 (3.4%)	*p*:0.414 *
	**VSD repair**	0 (0.0%)	0 (0.0%)	3 (2.4%)	0 (0.0%)	3 (0.8%)	*p*:0.383 *
**CPB time in minutes**		120.7 ± 34.4	116.9 ± 36.1	134.3 ± 36.8	128.2 ± 46.2	125.3 ± 37.4	*p*:0.001 *
**Cross clamp time in minutes**		85.6 ± 26.8	79.2 ± 29.4	99.7 ± 28.5	90.8 ± 30.7	89.7 ± 29.1	*p*:0.001 *
**Inotrope requirement**		81 (51.9%)	30 (50.0%)	85 (69.7%)	22 (53.7%)	218 (57.5%)	*p*:0.012 **
**Total follow-up time (months)**		41.1 ± 21.5	40.8 ± 20.6	27.3 ± 12.1	31.7 ± 14.4	35.6 ± 19.1	***p:0.001***
**First 30-day mortality**		13 (8.3%)	3 (5.0%)	1 (0.8%)	0 (0.0%)	17 (4.5%)	***p:0.011***
**Total mortality**		24 (15.4%)	7 (11.7%)	13 (10.7%)	3 (7.3%)	47 (12.4%)	*p*:0.453
**5-year survival**		132 (84.6%)	54 (90.0%)	109 (89.3%)	38 (92.7%)	333 (87.9%)	*p*:0.400

ASD: Atrial septal defect, VSD: Ventricular septal defect, CPB: Cardiopulmonary bypass

*Kruskal-Wallis test **Chi-Square test

**Table 3:** Comparison of the groups regarding stay time in the intensive care unit, postoperative hospitalization and complications

PARAMETERS		Group-1 (*n* = 156)	Group-2 (*n* = 60)	Group-3 (*n* = 122)	Group-4 (*n* = 41)	Total (*n* = 379)	Test Statistics
**Stay time in the ICU** **(days)**		3.0 ± 6.3 (1;0–43)	2.7 ± 5.6 (1;1–32)	2.7 ± 7.4 (1;1–54)	1.4 ± 0.7 (1;1–4)	2.7 ± 6.2 (1;0–54)	*p*:0.675 *
**Postoperative** **stay time (days)**		10.1 ± 10.7 (7;0–77)	9.5 ± 8.9 (7;5–64)	9.9 ± 12.2 (7;2-108)	7.8 ± 2.4 (7;5–17)	9.7 ± 10.4 (7;0-108)	*p*:0.761 *
**Postoperative Complications**	**Bleeding (n)**	6 (3.8%)	1 (1.7%)	4 (3.3%)	2 (4.9%)	13 (3.4%)	*p*:0.265 **
	**Tamponade(n)**	11 (7.1%)	1 (1.7%)	2 (1.6%)	2 (4.9%)	16 (4.2%)	
	**CVE (n)**	4 (2.6%)	0 (0.0%)	2 (1.6%)	0 (0.0%)	6 (1.6%)	
	**Cardiac Arrest (n)**	5 (3.2%)	2 (3.3%)	0 (0.0%)	0 (0.0%)	7 (1.8%)	
	**Sepsis (n)**	1 (0.6%)	1 (1.7%)	0 (0.0%)	0 (0.0%)	2 (0.5%)	
	**Total (n)**	27 (17.3%)	5 (8.3%)	8 (6.6%)	4 (9.8%)	44 (11.6%)	

ICU: Intensive Care Unit, CVE: Cerebrovascular Event

*Kruskal-Wallis test **Chi-Square test

### Postoperative length of stay and complications

Table 3 presents the length of stay in the Intensive Care Unit (ICU) and hospital during the postoperative period. The data indicated no statistically significant difference in the length of stay in the ICU and hospital among the groups (*p*: 0.675, *p*: 0.761, respectively).

### Postoperative complications

Complications, including bleeding, tamponade, cerebrovascular events (SVE), cardiac arrest, and sepsis, were observed in 44 patients (11.6%) during the postoperative period. However, there was no statistically significant difference in the incidence of these complications among the different operation groups (*p*: 0.265) (Table 3).

**Table 4:** Postoperative short-term TR, EF, SPAP data. Comparison of preoperative and postoperative short-term TR changes data

Parameters		Group-1 (*n* = 141)	Group-2 (*n* = 59)	Group-3 (*n* = 121)	Group-4 (*n* = 41)	Total (*n* = 362)	Test Statistics
**Postoperative short-term EF (%)**		50.8 ± 7.8	49.5 ± 8.0	49.7 ± 9.9	51.2 ± 8.3	50.3 ± 8.6	*p*:0.620 *
**Postoperative short-term SPAP (mmHg)**		40.4 ± 6.1	39.1 ± 6.4	35.9 ± 5.8	39.1 ± 5.9	38.5 ± 6.3	p:0.001 *
**Postoperative short-term TR (Mean ± Std. Deviation)**		2.6 ± 0.7	2.9 ± 0.9	1.6 ± 0.6	1.8 ± 0.6	2.2 ± 0.9	p:0.001**
**Preoperative - Postoperative short-term TR Comparison**	**Regression**	47 (24.2%)	15 (%7.7)	96 (49.5%)	36 (18.6%)	194 (53.6%)	p:0.001 **
	**Same**	68 (48.2%)	24 (%20.3)	21 (17.4%)	5 (4.2%)	118 (32.6%)	
	**Increase**	26 (18.5%)	20 (%33.9)	4 (3.3%)	0 (0.0%)	50 (13.8%)	

EF: Ejection Function; SPAP: Systolic Pulmonary Artery Pressure, TR: Tricuspid valve regurgitation, Mean ± Std: Mean, Standard Deviation NYHA: New York Heart Association Functional Capacity Classification

*Kruskal-Wallis test **Chi-Square test ^a^: Patient Distribution after Mortality

### EF and SPAP analysis

Tables 4 and 5 also present the short and long-term mean EF and SPAP for the four groups that underwent FTR operation. There was no statistically significant difference among the groups’ short-term EF averages (*p*: 0.620) (Table 4). However, regarding long-term EF averages, Group 3 and Group 4 exhibited significantly higher EF values than those in the other groups (*p*: 0.007) (Table 5). Regarding SPAP averages, the analysis of postoperative short and long-term values revealed that the SPAP averages of Group-3 patients were significantly lower than those in the other groups in the early postoperative period (*p*: 0.001) (Table 4). When examining the long-term SPAP averages, Group 3 and Group 4 showed similar values, significantly lower than the SPAP averages of Group 1 and Group 2 (*p*: 0.001) (Table 5).

**Table 5:** Postoperative long-term TR, EF, SPAP data. Comparison of postoperative long-term results with preoperative and postoperative short-term results

Parameters		Group-1 (*n* = 139)^a^	Group-2 (*n* = 57)^a^	Group-3 (*n* = 115)^a^	Group-4 (*n* = 40)^a^	Total (*n* = 351)^a^	Test Statistics
**Postoperative long-term EF (%)**		50.2 ± 8.0	48.5 ± 7.4	51.0 ± 8.3	52.3 ± 9.8	50.4 ± 8.3	p:0.007 *
**Postoperative long-term SPAP (mmHg)**		40.3 ± 6.9	40.6 ± 6.4	34.1 ± 6.0	35.5 ± 5.6	37.8 ± 7.0	p:0.001 *
**Postoperative long-term TR (Mean ± Std. Deviation)**		2.5 ± 0.9	2.7 ± 0.7	1.5 ± 0.6	1.6 ± 0.7	2.1 ± 0.9	p:0.001 **
**Postoperative short and long-term TR Comparison**	**Regression**	37 (26.6%)	18 (22.5%)	17 (14.8%)	8 (10.0%)	80 (22.8%)	p:0.001 **
	**Same**	75 (33.5%)	28 (12.5%)	90 (40.2%)	31 (13.8%)	224 (63.8%)	
	**Increase**	27 (19.4%)	11 (19.3%)	8 (7.0%)	1 (2.1%)	47 (13.4%)	
**Preoperative and Postoperative long-term NYHA** **Comparison**	**Regression**	75 (33.3%)	15 (6.7%)	99 (44.0%)	36 (16.0%)	225 (64.1%)	p:0.001 **
	**Same**	54 (38.8%)	33 (31.1%)	16 (13.9%)	3 (2.8%)	106 (30.2%)	
	**Increase**	10 (7.2%)	9 (15.8%)	0 (0.0%)	1 (2.5%)	20 (5.7%)	

EF: Ejection Function; SPAP: Systolic Pulmonary Artery Pressure TR: Tricuspid valve regurgitation, Mean ± Std: Mean, Standard Deviation NYHA: New York Heart Association Functional Capacity Classification

*Kruskal-Wallis test **Chi-Square test ^a^: Patient Distribution after Mortality

### Changes in tricuspid valve status

Patients were compared based on the changes in tricuspid valve status during the preoperative, early postoperative, and late postoperative periods. The tricuspid valve regurgitation (TR) changes were categorized as regression, unchanged, or progression (Tables 4 and 5).

Comparing the preoperative period with the early postoperative period, Group 3 and Group 4 had the highest rates of TR regression, while in Group 1, the rate of change was the lowest. Notably, Group 4 did not show any increase in TR compared to the preoperative period, whereas Group 1 and Group 2 exhibited the highest increase. These findings were found to be statistically significant (*p*: 0.001) (Table 4).

When comparing the early postoperative period with the late postoperative period, Group 1 had the highest rate of TI regression. There was no significant difference between Group 3 and Group 4 in terms of TI remaining the same, while Group 1 and Group 2 showed a more significant proportion. The highest increase was observed in Group 1 and Group 2. These findings were considered statistically significant (*p*: 0.001) (Table 5).

### Postoperative long-term NYHA status

Table 5 illustrates the postoperative long-term status of patients based on the New York Heart Association (NYHA) functional capacity classification, indicating regression, no change, or progression compared to the preoperative period. Overall, a regression rate of 64.1% was observed in all groups, with higher regression rates in Group 3 and Group 4 compared to those in the other groups. Conversely, Group 1 and Group 2 had a higher proportion of patients who remained unchanged. Notably, no increase was observed in Group 3, but the majority of patients with an increase were found in Group 1 and Group 2. These findings were statistically significant (*p*: 0.001).

## Discussion

Tricuspid valve regurgitation (TR) is a prevalent condition often associated with left heart pathologies, particularly termed Functional Tricuspid Valve Regurgitation (FTR) when linked to right heart dilation, afterload, and preload. Current consensus and guidelines strongly advocate for addressing the tricuspid valve during surgery for the primary pathology [9]. While tricuspid valve repair has become the preferred surgical approach for FTR, valve replacement is generally recommended for cases of organic tricuspid regurgitation [10]. FTR patients undergoing tricuspid valve repair, as demonstrated in a study by Kevin et al., have shown acceptable short- and long-term mortality rates [11]. Similarly, our study, consistent with previous findings, did not identify significant mortality rates during the 5-year follow-up of patients who had undergone surgery for FTR.

A primary concern for patients undergoing valve repair for FTR is the potential persistence of FTR in the future. The degree of residual tricuspid regurgitation and its impact on clinical outcomes play a crucial role. Brescia et al. reported in a 14-month follow-up study that although residual tricuspid regurgitation was observed in patients who had undergone valve repair for FTR, the occurrence of moderate to severe regurgitation was minimal [12]. Similarly, in our study, while residual tricuspid regurgitation was detected in patients who had undergone valve repair, there was a significant reduction in cases with severe regurgitation following the operation.

Since the recognition of the need to repair FTR, various repair methods have been developed. The annular plication technique, known as the Kay method, was one of the initial approaches in FTR operations, involving leaving the posterior leaflet outside and creating a bicuspid tricuspid valve [13]. However, due to unsatisfactory long-term outcomes of Kay suture annuloplasty, new methods, such as the De Vega technique introduced by De Vega, have emerged. The De Vega technique entails narrowing the dilated annulus around the anterior and septal leaflets using purse-string sutures [14]. Carpentier introduced the first rigid ring annuloplasty in 1974, two years after the De Vega technique [15]. Over time, annuloplasty rings have become more diverse, including semi-rigid, flexible, and biodegradable rings, better accommodating the annulus’s nature and allowing for movement.

In a study conducted by Hata et al., different ring annuloplasty methods did not show a significant difference in long-term survival, but ring annuloplasty was advantageous in reducing the degree of FTR based on the mean TR degree [16]. Furthermore, a meta-analysis conducted by Di Mauro et al. analyzed data from 31 studies involving 9,663 patients who had undergone surgery for FTR. The analysis aimed to comprehensively evaluate outcomes across studies. The results indicated that the ring annuloplasty method for FTR demonstrated superior survival outcomes compared to the suture annuloplasty method or the option of not operating on the tricuspid valve [17]. Another study examined the long-term effects of the Rjit ring and De Vega annuloplasty in FTR operations, yielding varying findings regarding survival. While the type of operation did not significantly impact survival, rigid ring annuloplasty showed potential benefits in reducing tricuspid regurgitation severity in the long term. Both rigid and flexible annuloplasty techniques were effective in FTR operations, with the rigid ring annuloplasty technique associated with lower tricuspid regurgitation severity [11]. In the present study, the 30-day mortality rate was significantly higher in Group 1 compared to other techniques in FTR operations. However, there was no difference in 5-year survival rates among the groups.

In a study conducted by Fang et al., 148 patients with FTR were analyzed and divided into three groups: 58 patients underwent the Cosgrove-Edward technique, 62 patients underwent the Kay technique, and 29 patients underwent the De Vega technique. After a mean follow-up of 7 months, all groups showed significant regression in the degree of tricuspid regurgitation compared to the preoperative period. Similarly, there was a significant improvement in NYHA functional class values postoperatively compared to preoperative values. The study indicated that the group receiving annuloplasty showed statistically superior outcomes in these parameters compared to the other techniques [18]. A study by Navia et al. analyzed 2,277 patients who had undergone tricuspid valve operation in conjunction with mitral and aortic valve operations. The study revealed that a rigid tricuspid annular ring was used in 26% of patients, a flexible ring in 46% of patients, the De Vega technique in 5.7% of patients, Peri-Guard in 8.1% of patients, the Kay method in 11% of patients, and the end-to-end leaflet suture technique in 3.5% of patients. After a 5-year follow-up, it was reported that rigid and semi-rigid annuloplasty techniques were superior in preventing recurrent or progressive tricuspid regurgitation [19].

Our study observed a significant decrease in tricuspid regurgitation and improvement in NYHA functional capacity in the postoperative period across all surgical methods compared to preoperative values. When comparing annuloplasty methods in terms of tricuspid regurgitation, SPAP, and NYHA functional capacity changes, the parameters were statistically significantly more favorable in patients who had undergone ring annuloplasty compared to those who had undergone suture annuloplasty.

## Conclusion

In conclusion, ring annuloplasty techniques have demonstrated superiority over other methods in reducing SPAP, regressing TR, and improving NYHA functional capacity in repairs of FTR. Whether utilizing flexible or semi-rigid rings, tricuspid ring annuloplasty proves to be an effective and safe approach for tricuspid valve repairs, with no significant increase in mortality or morbidity.

## Data Availability

The article’s data will be shared upon request with the corresponding author.

## Abbreviations

FTR: Functional tricuspid valve regurgitation
SPAP: Systolic pulmonary artery pressure
CPB: Cardio-Pulmonary Bypass
TR: Tricuspid valve regurgitation
NYHA: New York Heart Association
EF: Ejection fraction
MVR: Mitral valve replacement
AVR: Aortic valve replacement
ASD: Atrial septal defect
CABG: Coronary artery bypass grafting
VSD: Ventricular septal defect
CVE: Cerebrovascular events
ICU: Intensive Care Unit
HT: Hypertension
DM: Diabetes mellitus
COPD: Chronic obstructive pulmonary disease
CRF: Chronic renal failure
CMC: Closed mitral commissurotomy
ECHO: Echocardiography
MVS: Mitral valve stenosis
MVI: Mitral valve insufficiency
